# The impact of oxygen on the final alcohol content of wine fermented by a mixed starter culture

**DOI:** 10.1007/s00253-014-6321-3

**Published:** 2015-01-13

**Authors:** Pilar Morales, Virginia Rojas, Manuel Quirós, Ramon Gonzalez

**Affiliations:** 1 Instituto de Ciencias de la Vid y del Vino (CSIC, Universidad de la Rioja, Gobierno de La Rioja), Logroño, La Rioja Spain; 2 Facultad de Ciencias Químicas, Universidad de Autónoma de San Luis Potosí, Centro Histórico, Mexico; 3 Present Address: Evolva Biotech A/S, Copenhagen Ø, Denmark

**Keywords:** Low alcohol, Non-*Saccharomyces* yeast, Respiratory quotient, Dissolved oxygen, Volatile acidity

## Abstract

We have developed a wine fermentation procedure that takes advantage of the metabolic features of a previously characterized *Metschnikowia pulcherrima* strain in order to reduce ethanol production. It involves the use of *M. pulcherrima*/*Saccharomyces cerevisiae* mixed cultures, controlled oxygenation conditions during the first 48 h of fermentation, and anaerobic conditions thereafter. The influence of different oxygenation regimes and initial inoculum composition on yeast physiology and final ethanol content was studied. The impact of oxygenation on yeast physiology goes beyond the first aerated step and influences yields and survival rates during the anaerobic stage. The activity of *M. pulcherrima* in mixed oxygenated cultures resulted in a clear reduction in ethanol yield, as compared to *S. cerevisiae*. Despite relatively low initial cell numbers, *S. cerevisiae* always predominated in mixed cultures by the end of the fermentation process. Strain replacement was faster under low oxygenation levels. *M. pulcherrima* confers an additional advantage in terms of dissolved oxygen, which drops to zero after a few hours of culture, even under highly aerated conditions, and this holds true for mixed cultures. Alcohol reduction values about 3.7 % (*v*/*v*) were obtained for mixed cultures under high aeration, but they were associated to unacceptable volatile acidity levels. In contrast, under optimized conditions, only 0.35 g/L acetic acid was produced, for an alcohol reduction of 2.2 % (*v*/*v*), and almost null dissolved oxygen during the process.

**Electronic supplementary material:**

The online version of this article (doi:10.1007/s00253-014-6321-3) contains supplementary material, which is available to authorized users.

## Introduction

Consumer preferences toward well-structured, full body wines have driven the requirement for late harvests in order to ensure an optimal phenolic maturity of grapes. In the context of global warming, this practice results in a noticeable increase in the sugar content of the berries at harvest (Mira de Orduña 2010) that finally gives rise to higher alcohol levels in wine. This leads to numerous wine quality, marketing, and public health issues. In order to compensate for this increase, the wine industry has been seeking for new approaches to reduce the alcohol content of wines. Several technological solutions including winemaking practices adapted to unripe berries (Kontoudakis et al. 2011; Canals et al. 2008) or partial dealcoholization by physical methods (Schmidtke et al. 2012; Catarino and Mendes 2011; Belisario-Sánchez et al. 2009) have been proposed to this end. Additionally, several research articles have focused on unconventional microbiological solutions for this purpose. Among them, the development of low alcohol yield yeast strains has been, and still is, a hot topic in the field of winemaking. In this context, different metabolic or evolutionary engineering strategies aiming to divert the carbon flux from ethanol production in *Saccharomyces cerevisiae* have been proposed (Michnick et al. 1997; Cadiere et al. 2011; Heux et al. 2006; Rossouw et al. 2013; Varela et al. 2012a). The topic has been extensively reviewed by Kutyna et al. (2010). However, modifying ethanol yields in this species has been proven to be a difficult task, mainly due to the tight regulation of the pyruvate node under anaerobic conditions (Varela et al. 2004; Quirós et al. 2013). Consequently, limited success has been generally achieved. Additionally, the industrial application of most of these approaches is currently limited by the concomitant overproduction of non-desired metabolites such as acetate, acetaldehyde or acetoin (Heux et al. 2006), public attitudes toward genetically modified organisms (GMOs) and/or regulations that restrict their usefulness in the wine industry. Recent reports indicate that experimental evolution might be a feasible alternative to genetic engineering in order to develop *S. cerevisiae* yeast strains with reduced alcohol yield (Tilloy et al. 2014).

While *S. cerevisiae* is the main yeast species responsible for conducting the alcoholic fermentation of grape must, the contribution of a non-negligible number of other yeast species to the initial stages of the process and to the sensorial properties of wine is currently well established (Fleet 2003; Medina et al. 2013; Ciani et al. 2010; Rojas et al. 2003; Sadoudi et al. 2012; Cordero-Bueso et al. 2013). These species, naturally present in sound grapes, are mainly represented by strains belonging to the apiculate yeast genus *Hanseniaspora* (mainly *Hanseniaspora uvarum* or its anamorph *Kloeckera apiculata*) and other species of the genera *Candida*, *Pichia*, *Kluyveromyces*, and *Metschnikowia* (Fleet 2007; Tamang and Fleet 2009).

Our research group recently proposed the possibility of using non-*Saccharomyces* yeast species for the reduction of the alcohol content of wine (Gonzalez et al. 2013). Key differences in sugar metabolism between some of these species and *S. cerevisiae* could actually allow for an increased breakdown of sugars via respiratory pathways rather than through fermentation, provided that an appropriate amount of oxygen is available. The possibility of using respiratory catabolism as a clean way to limit sugar conversion to ethanol had been previously suggested by other authors (Smith 1995; Erten and Campbell 2001; Bärwald and Fischer 1996). However, most of these studies describe preliminary results and went almost unnoticed for several reasons, including limited availability of the original documents (Smith 1995), limitations in the experimental setup, or low number of yeast strains screened. A Crabtree-negative recombinant *S. cerevisiae* wine yeast strain derivative developed by Henricsson et al. (2005) would also be interesting in this context. However, commercial application of such strain would experience the inconveniences associated to its GMO status.

In a recent work, our group surveyed around 60 non-*Saccharomyces* yeast strains of 29 different species to evaluate their potential application as starter cultures for lowering the ethanol content of wines (Quirós et al. 2014). This study, which set the focus on yeast key physiological parameters during the aerobic metabolism of synthetic must, concluded that high acetic acid yields constituted the main handicap for this application (Quirós et al. 2014). It was also found that high dissolved oxygen (DO) levels were not required for relevant yeast respiration (Quirós et al. 2014). Among the yeast species studied, different isolates belonging to the species *Metschnikowia pulcherrima* stood out due to their low ethanol and acetate yields on sugar and high sugar conversion rate. The usefulness of this non-conventional species for the aforementioned purpose was also remarked by Contreras et al. (2014), where a reduction in the alcohol level between 0.9 and 1.6 % (*v*/*v*) was achieved in fermentations performed by sequential inoculation. Nevertheless, the potential contribution of respiration to alcohol reduction was not explored or discussed in that study.

As a proof of concept, we addressed the effect of different aeration conditions and different co-inoculation ratios of *S. cerevisiae* and *M. pulcherrima* strains selected from our previous work (Quirós et al. 2014), with the aim of achieving a significant reduction of the alcohol level of wine, while limiting volatile acidity production and the contact of grape must components with molecular oxygen.

## Materials and methods

### Yeast strains

A commercial *S. cerevisiae* wine yeast strain, EC1118 (Lallemand Inc., Montreal, Canada), and *M. pulcherrima* CECT12841, selected from a previous study (Quirós et al. 2014). The strains were grown at 28 °C and maintained at 4 °C on yeast peptone dextrose (YPD) plates (2 % glucose, 2 % peptone, 1 % yeast extract, and 2 % agar), as well as in glycerol stocks at −80 °C.

### Controlled aeration fermentation assays

Fermentation experiments were performed in triplicate (for cultures sparged with pure air or nitrogen) or duplicate (for intermediate aeration conditions), using MiniBio bioreactors (250 mL nominal volume) equipped with Peltier-refrigerated gas condensers (Applikon Biotechnology B.V., Delft, The Netherlands). Seed cultures were grown in YPD broth for 48 h, at 25 °C and 250 rpm. Bioreactors were filled with 150 mL of a filter-sterilized natural white grape must, a mixture of Malvasia and Viura varieties with no added carbon or nitrogen sources (262–265 g/L of sugars; 200 mg/L yeast assimilable nitrogen), and 200 μL (approx.) of antifoam 204 (Sigma-Aldrich, St. Louis, MO). The same batch of grape must was used for all experiments. Temperature was set to 25 °C, initial stirring to 1000 rpm, and inoculation to approximately 0.2 initial optical density at 600 nm (OD_600_). Initial proportions of both strains in co-inoculation experiments were based on OD_600_ values. The cultures were sparged in a discontinuous regime with pure N_2_, pure air, or mixtures of both, at a gas flow rate of 3.0 L/h (i.e. 20 gas volumes/culture volume/h (vvh)). Gas flow was controlled with MFC17 mass flow controllers (Aalborg Instruments and Controls, Inc., Orangeburg, NY), whose calibration was regularly verified with a soap bubble flow meter. In a preliminary assay, aeration was automatically controlled from time zero in order to maintain a DO level above 15 % (with air) in the pure culture of *M. pulcherrima* CECT12841. Sparging was totally interrupted 48 h after inoculation, and stirring slowed down to 200 rpm. Independently of the gas used and in order to improve reproducibility of the experiments, as well as to standardize any possible effect on the loss of volatile compounds, successive experiments were performed using a programmed on/off pattern for the gas valve, mimicking the pattern obtained in that preliminary experiment (Table S1 in the Supplementary Material). Samples for determination of metabolite concentrations were withdrawn every 12 h for the first 2 days and every 24 h thereafter. Population dynamics was monitored every 24 h.

### Viable counts in pure and mixed cultures

Evolution of global biomass in all fermentation assays was monitored by daily determination of OD_600_. Viable cells for each of the strains were quantified by plating appropriate dilutions in YPD plates and incubating for 48–72 h at 25 °C. Colonies of *S. cerevisiae* EC1118 in mixed cultures were distinguished from those of *M. pulcherrima* CECT12841 by the development of a pink coloration by the latter.

### Determination of metabolite concentrations

The concentration of glucose, fructose, glycerol, ethanol, and acetic acid was determined using a Surveyor Plus Liquid Chromatograph (Thermo Fisher Scientific, Waltham, MA) equipped with a refraction index and a photodiode array detector (Surveyor RI Plus and Surveyor PDA Plus, respectively) on a 300 × 7.7 mm HyperREZ^TM^ XP Carbohydrate H+ (8 μm particle size) column and guard (Thermo Fisher Scientific). The column was maintained at 50 °C, and 1.5 mM H_2_SO_4_ was used as the mobile phase at a flow rate of 0.6 mL/min. Prior to injection in duplicate, the samples were filtered through 0.22 μm pore size nylon filters (Micron Analitica, Madrid, Spain) and diluted 10-fold in MilliQ water.

Yeast assimilable nitrogen in natural grape must was determined spectrophotometrically as the sum of the contributions of free ammonium and free amino groups. Ammonium was assayed using a specific R-Biopharm assay kit (Darmstadt, Germany). Free amino groups were determined with *ο*-phthaldialdehyde (Dukes and Butzke 1998).

### Statistical analysis

One way analysis of variance was carried out on the main fermentation metabolites found on day 2 and on finished fermentations sparged with pure air or nitrogen, with inoculum composition as main effect on each aeration condition. The effect of aeration on the main fermentation metabolites was also analyzed by means of one-way ANOVA on each inoculum composition. Means were compared using Tukey’s test, with significance level set at 5 %. Data from fermentations sparged with gas mixtures (10 % or 25 % air) were compared by Student’s *t* test with significance level set at 5 %. Correlation between main yields and air content in the sparging gas was analyzed by Pearson correlation analysis. All analyses were performed using SPSS Statistics v. 20 program (IBM, Armonk, NY).

## Results

To establish the aeration regime for all further experiments in this study, we performed an initial assay with *M. pulcherrima* CECT12841 in natural grape must. The gas valve control was set to open every time DO level fell below 15 % (with air) during the first 48 h. This saturation level was taken as a suitable balance between the precision of oxygen saturation measurement and the expected yeast requirements for respiration. According to data from Saa et al. (2012) for oxygen solubility in fermentation media at 25 °C (i.e. about 7 mg/L), 15 % oxygen saturation (with air) would be equivalent to around 1 mg/L of molecular oxygen. The records of the experiment indicated the total opening time for the air valve that was about 30 h. This information, as well as the timing of the actuator status (on/off), was used to design the gassing pattern employed in the subsequent experiments (Table S1 in the Supplementary Material). In order to minimize any ambiguity in the interpretation of the analytical results that could have arisen from variations in the gassing regimes (mainly concerning ethanol stripping), all the experiments analyzed in this work were sparged following the above-mentioned pattern and with exactly the same gas flow (20 vvh), independently of the composition of the sparging gas.

We had previously demonstrated respiratory metabolism for *M. pulcherrima* CECT12841, under similar conditions as described in “Materials and methods” with lower but continuous air flow (1.2 L/h). Indeed respiratory quotient (RQ) values remained close to 1 (indicating pure respiration) until dissolved oxygen levels were undetectable (Quirós et al. 2014). From that point, a steady increase in RQ values was observed. *S. cerevisiae* EC1118 also showed respiro-fermentative metabolism under such conditions but always with higher RQ values than *M. pulcherrima* (Quirós et al. 2014).

### Impact of *M. pulcherrima* CECT12841 and aeration on the initial yield of the main fermentation metabolites

Initial assays were performed using either air or nitrogen as sparging gas and four different strain combinations: pure cultures of *S. cerevisiae* EC1118 or *M. pulcherrima* CECT12841, and mixed cultures with the same inoculation level of *M. pulcherrima*, plus 1 or 10 % the inoculation level of *S. cerevisiae* used in the pure culture.

Concentration and yield of the main fermentation metabolites during the aerated step (first 48 h of culture) are shown in Table 1. For *S. cerevisiae*, a clear impact of aeration is observed on the yield of glycerol, ethanol, and acetic acid. Glycerol and ethanol yields decreased with air, while acetic acid yield increased by more than five times (Table 1).

**Table 1:** Concentration and yields of the main fermentation metabolites for fermentations sparged with air or nitrogen, in the conditions described in the text, after 48 h of culture (end of the aerated step

		*S. cerevisiae* ^a^	*M. pulcherrima* + 10 % *S. cerevisiae* ^a^	*M. pulcherrima* + 1 % *S. cerevisiae* ^a^	*M. pulcherrima* ^a^
Glycerol (% *w*/*v*)	Air^b^	0.69 ± 0.03*A	1.23 ± 0.15B	1.21 ± 0.04*B	1.18 ± 0.17B
	Nitrogen^b^	0.93 ± 0.08*	1.05 ± 0.03	1.09 ± 0.06*	1.05 ± 0.11
Ethanol (% *v*/*v*)	Air^b^	5.1 ± 0.2*B	1.8 ± 0.5*A	2.0 ± 0.5*A	2.6 ± 1.5A
	Nitrogen^b^	6.7 ± 0.5*C	5.1 ± 0.1*B	4.0 ± 0.1*A	3.2 ± 0.5A
Acetic acid (mg/L)	Air^b^	878 ± 62*B	93 ± 43A	86 ± 31A	83 ± 25A
	Nitrogen	165 ± 125*	64 ± 16	58 ± 5	45 ± 11
Cons. sugars (% *w*/*v*)	Air^b^	12.9 ± 0.7B	9.6 ± 0.8A	9.6 ± 0.8A	10.1 ± 1.2*A
	Nitrogen^b^	13.2 ± 0.9C	10.8 ± 0.8B	8.8 ± 1.0AB	7.1 ± 0.6*A
Y_E/S_ (g/g)	Air^b^	0.316 ± 0.005*B	0.150 ± 0.032*A	0.165 ± 0.025*A	0.197 ± 0.091*AB
	Nitrogen	0.399 ± 0.001*	0.375 ± 0.030*	0.362 ± 0.043*	0.357 ± 0.040*
Y_A/S_ (mg/g)	Air^b^	6.831 ± 0.523*B	0.953 ± 0.394A	0.883 ± 0.241A	0.821 ± 0.227A
	Nitrogen	1.218 ± 0.831*	0.592 ± 0.116	0.670 ± 0.112	0.641 ± 0.207
Y_G/S_ (g/g)	Air^b^	0.054 ± 0.001*A	0.129 ± 0.028B	0.127 ± 0.014B	0.120 ± 0.031B
	Nitrogen^b^	0.070 ± 0.001*A	0.098 ± 0.010AB	0.125 ± 0.019BC	0.148 ± 0.014C

Values are shown as mean ± standard deviation of three biological replicates.

*Y*_*E/S*_ ethanol yield on sugar, *Y*_*A/S*_ acetic acid yield on sugar, *Y*_*G/S*_ glycerol yield on sugar

^a^Statistically significant differences (ANOVA) between cultures sparged with air or nitrogen for the same parameter and inoculum are indicated by *

^b^Different capital letters indicate statistically significant differences (ANOVA) for values in the same row

*M. pulcherrima* responded differently to aeration (Table 1). On one side, sugar consumption clearly increased with aeration. On the other side, ethanol yield decreased to a higher extent than observed for *S. cerevisiae* (from 0.357 to 0.197 g/g). Finally, under air sparging conditions, *M. pulcherrima* cultures showed a lower acetic acid yield and higher glycerol yield than *S. cerevisiae* (Table 1).

Mixed cultures showed (i) lower sugar consumption values than those of *S. cerevisiae* under both aerobic and anaerobic conditions; (ii) similar acetic acid yields to those of *M. pulcherrima*, with little impact of oxygen availability (resulting in values clearly lower than *S. cerevisiae* under these conditions); and (iii) a large reduction in ethanol yield when sparged with air, down to values about half those of *S. cerevisiae* (Table 1). Up to this point, no statistically significant differences were found between cultures inoculated with 1 or 10 % *S. cerevisiae* (Table 1).

### Long-term impact of initial aeration

After 11 days, sugars were totally consumed, both for *S. cerevisiae* and mixed cultures. However, fermentations with pure *M. pulcherrima* cultures were sluggish, with more than 50 g/L residual sugar at this time point (data not shown).

By the end of the fermentation, a clear increase in ethanol yield was observed for all cultures (Table 2), as compared to data for the first 2 days.

**Table 2:** Concentration and yields of the main fermentation metabolites by the end (262–265 g/L sugar consumed) of fermentations sparged with air or nitrogen in the conditions described in the text

		*S. cerevisiae* ^a^	*M. pulcherrima* + 10 % *S. cerevisiae* ^a^	*M. pulcherrima* + 1 % *S. cerevisiae* ^a^
Glycerol (% *w*/*v*)	Air^b^	0.83 ± 0.02*A	1.86 ± 0.18*B	1.79 ± 0.06B
	Nitrogen^b^	1.20 ± 0.04*A	1.46 ± 0.06*B	1.65 ± 0.06C
Ethanol (% *v*/*v*)	Air^b^	12.9 ± 0.2*B	11.0 ± 0.3*A	11.1 ± 0.2*A
	Nitrogen^b^	14.7 ± 0.2*	13.9 ± 0.6*	13.9 ± 0.4*
Acetic acid (mg/L)	Air^b^	2158 ± 329*B	676 ± 63*A	682 ± 123*A
	Nitrogen	185 ± 47*B	63 ± 3*A	62 ± 2*A
Y_E/S_ (g/g)	Air^b^	0.384 ± 0.007*B	0.329 ± 0.010*A	0.330 ± 0.006*A
	Nitrogen^b^	0.441 ± 0.006*	0.417 ± 0.014*	0.416 ± 0.010*
Y_A/S_ (mg/g)	Air^b^	8.159 ± 1.241*B	2.553 ± 0.237*A	2.579 ± 0.461*A
	Nitrogen	0.703 ± 0.178*B	0.238 ± 0.010*A	0.236 ± 0.007*A
Y_G/S_ (g/g)	Air^b^	0.031 ± 0.001*A	0.070 ± 0.007*B	0.067 ± 0.002B
	Nitrogen^b^	0.045 ± 0.001*A	0.055 ± 0.002*B	0.063 ± 0.002C

Values are shown as mean ± standard deviation of three biological replicates

Y_E/S_ ethanol yield on sugar, Y_A/S_ acetic acid yield on sugar, Y_G/S_ glycerol yield on sugar

^a^Statistically significant differences (ANOVA) between cultures sparged with air or nitrogen for the same parameter and inoculum are indicated by *

^b^Different capital letters indicate statistically significant differences (ANOVA) for values in the same row

Stage-specific yields on glucose for the non-aerated stage were calculated taking into account the sugar consumed and the increase in ethanol from day 2 to the end of fermentation. Ethanol yields on glucose for this stage ranged from 0.431 to 0.481 g/g (Table 3). Different stage-specific yields were also observed for acetic acid and glycerol as a function of both inoculum and sparging gas composition (Table 3).

**Table 3:** Anaerobic stage-specific yields calculated for the main fermentation metabolites after sparging was completely stopped (from day 2 to the end of fermentation)

		*S. cerevisiae* ^a^	*M. pulcherrima* + 10 % *S. cerevisiae* ^a^
Y_G/S_ (g/g)	Air^b^	0.010 ± 0.004*A	0.037 ± 0.000*B
	Nitrogen	0.020 ± 0.003*	0.026 ± 0.006*
Y_E/S_ (g/g)	Air	0.451 ± 0.023	0.431 ± 0.024
	Nitrogen	0.481 ± 0.007	0.446 ± 0.022
Y_A/S_ (mg/g)	Air	9.513 ± 2.796*B	3.454 ± 0.519*A
	Nitrogen	0.125 ± 0.619*	−0.010 ± 0.089*

Values are shown as mean ± standard deviation of three biological replicates

*Y*_*E/S*_ ethanol yield on sugar, *Y*_*A/S*_ acetic acid yield on sugar, *Y*_*G/S*_ glycerol yield on sugar

^a^Statistically significant differences (ANOVA) between cultures sparged with air or nitrogen for the same parameter and inoculum are indicated by *

^b^Different capital letters indicate statistically significant differences (ANOVA) for values in the same row

The lowest ethanol production values were observed for aerated fermentations. Reduction was of about 1.8 % (*v*/*v*) for *S. cerevisiae* and 2.8 % (*v*/*v*) for mixed cultures (Table 2), as compared to anaerobic fermentation. The alcohol level reduction obtained with aerated mixed cultures, compared to anaerobic fermentations with *S. cerevisiae*, was around 3.7 % (*v*/*v*) (11.0–11.1 vs 14.7 % ethanol, respectively) (Table 2).

Acetic acid production for mixed cultures was about one third that of *S. cerevisiae*, under either air or nitrogen sparging. Unfortunately, even for mixed cultures, the levels reached in aerated fermentations were above 0.65 g/L (Table 2), which would not be acceptable for most consumers or market regulations in different countries. Finally, the trend toward increased glycerol yield by mixed, aerated fermentations, already observed for 48 h samples, was confirmed as statistically significant by the end of fermentation (Table 2).

### Impact of different aeration levels

Two additional oxygenation levels were assayed with pure *S. cerevisiae* and mixed cultures (10 % *S. cerevisiae*). This was done by following the same gassing pattern as above but using gas mixtures containing 10 or 25 % air, which would result in maximum dissolved oxygen levels around 0.7 and 1.7 mg/L respectively. Data on main fermentation metabolites for these experiments are shown in Table 4. Considering data from the four oxygenation levels, a negative correlation between air concentration and ethanol yield was found for both inocula (Table 5). In addition, for each condition, ethanol yield was always lower for the mixed culture than for *S. cerevisiae* (Tables 2 and 4).

**Table 4:** Concentration and yields of the main fermentation metabolites by the end of fermentations sparged with air/nitrogen mixtures in the conditions described in the text

		Inoculum	
		*S. cerevisiae*	*M. pulcherrima* + 10 % *S. cerevisiae*
Glycerol (% *w*/*v*)	10 % Air^a^	1.06 ± 0.05A	1.21 ± 0.01B
	25 % Air^a^	0.97 ± 0.02A	1.11 ± 0.01B
Ethanol (% *v*/*v*)	10 % Air^a^	14.2 ± 0.0B	13.2 ± 0.1A
	25 % Air^a^	13.8 ± 0.1B	12.6 ± 0.2A
Acetic acid (mg/L)	10 % Air^a^	451 ± 71B	208 ± 3A
	25 % Air	1074 ± 267	351 ± 15
Y_E/S_ (g/g)	10 % Air^a^	0.425 ± 0.001B	0.394 ± 0.002A
	25 % Air^a^	0.412 ± 0.003B	0.377 ± 0.005A
Y_A/S_ (mg/g)	10 % Air^a^	1.706 ± 0.268B	0.787 ± 0.011A
	25 % Air	4.060 ± 1.008	1.326 ± 0.055
Y_G/S_ (g/g)	10 % Air^a^	0.040 ± 0.002A	0.046 ± 0.000B
	25 % Air	0.036 ± 0.001A	0.042 ± 0.001B

Values are shown as mean ± standard deviation of two biological replicates

*Y*_*E/S*_ ethanol yield on sugar, *Y*_*A/S*_ acetic acid yield on sugar, *Y*_*G/S*_ glycerol yield on sugar

^a^Different capital letters indicate statistically significant differences for values in the same row (*t* test)

**Table 5:** Correlation between the final yields of main fermentation metabolites and air concentration in the sparging gas for different inocula

	*S. cerevisiae*	*M. pulcherrima* + 10 % *S. cerevisiae*
Y_E/S_	−0.947**	−0.953**
Y_A/S_	0.962**	0.970**
Y_G/S_	−0.908**	0.754*

*Y*_*E/S*_ ethanol yield on sugar, *Y*_*A/S*_ acetic acid yield on sugar, *Y*_*G/S*_ glycerol yield on sugar

*Statistically significant at the 0.05 level

**Statistically significant at the 0.01 level

Positive correlations were confirmed between acetic acid yield and oxygenation level (Table 5). However, glycerol yields showed opposite trends when considering the pure *S. cerevisiae* cultures or the mixed ones. In addition, while the trend for *S. cerevisiae* pure cultures is clear, a J-shaped graph was obtained for the mixed cultures (not shown).

### Population dynamics

**Fig. 1:**
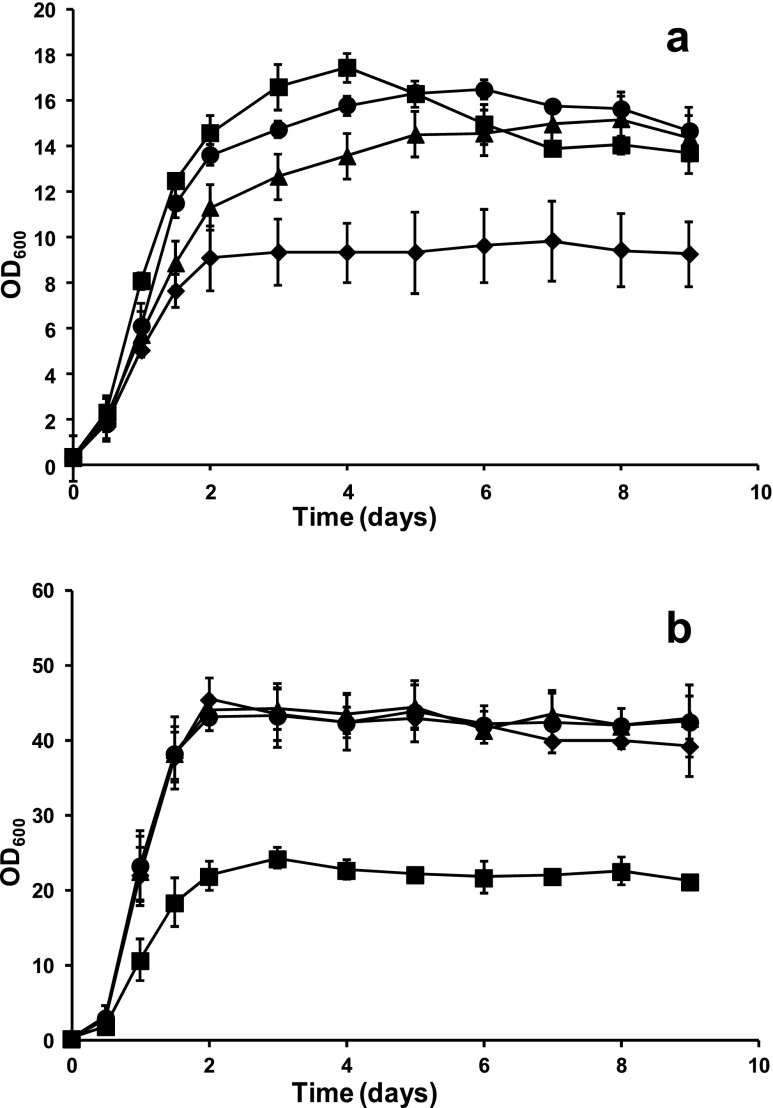
Cell growth in fermentations sparged with nitrogen (**a**) or air (**b**), in the conditions described in the text, for different combinations of yeast strains in the inoculums: pure *M. pulcherrima* (*diamonds*), pure *S. cerevisiae* (*squares*), *M. pulcherrima* plus 1 % *S. cerevisiae* (*triangles*), and *M. pulcherrima* plus 10 % *S. cerevisiae* (*circles*). Note that vertical axes are different between panels

Under all experimental conditions, cell growth took place mainly during the first 48 h of culture (Fig. 1). The highest biomass values were obtained in fermentation experiments sparged with air and inoculated with *M. pulcherrima* either alone or in mixed culture, reaching OD_600_ values close to 50 (Fig. 1). Under these conditions, OD_600_ values for *S. cerevisiae* pure cultures were about half those of pure *M. pulcherrima*. Cultures sparged with nitrogen reached much lower biomass values, as expected for pure fermentative metabolism. In addition, the relative advantages of *S. cerevisiae* and *M. pulcherrima* were inverted when cultures were sparged with nitrogen. Under these conditions, mixed and pure *S. cerevisiae* cultures reached OD_600_ values clearly higher than the *M. pulcherrima* pure culture (Fig. 1). In cultures sparged with air/nitrogen mixtures (10 or 25 % air), biomass production was closer to cultures sparged with air than to anaerobic cultures (data not shown). These results are in agreement with our previous demonstration of respiratory metabolism for *S. cerevisiae* EC1118 and *M. pulcherrima* CECT12841 under similar experimental conditions (Quirós et al. 2014).

**Fig. 2:**
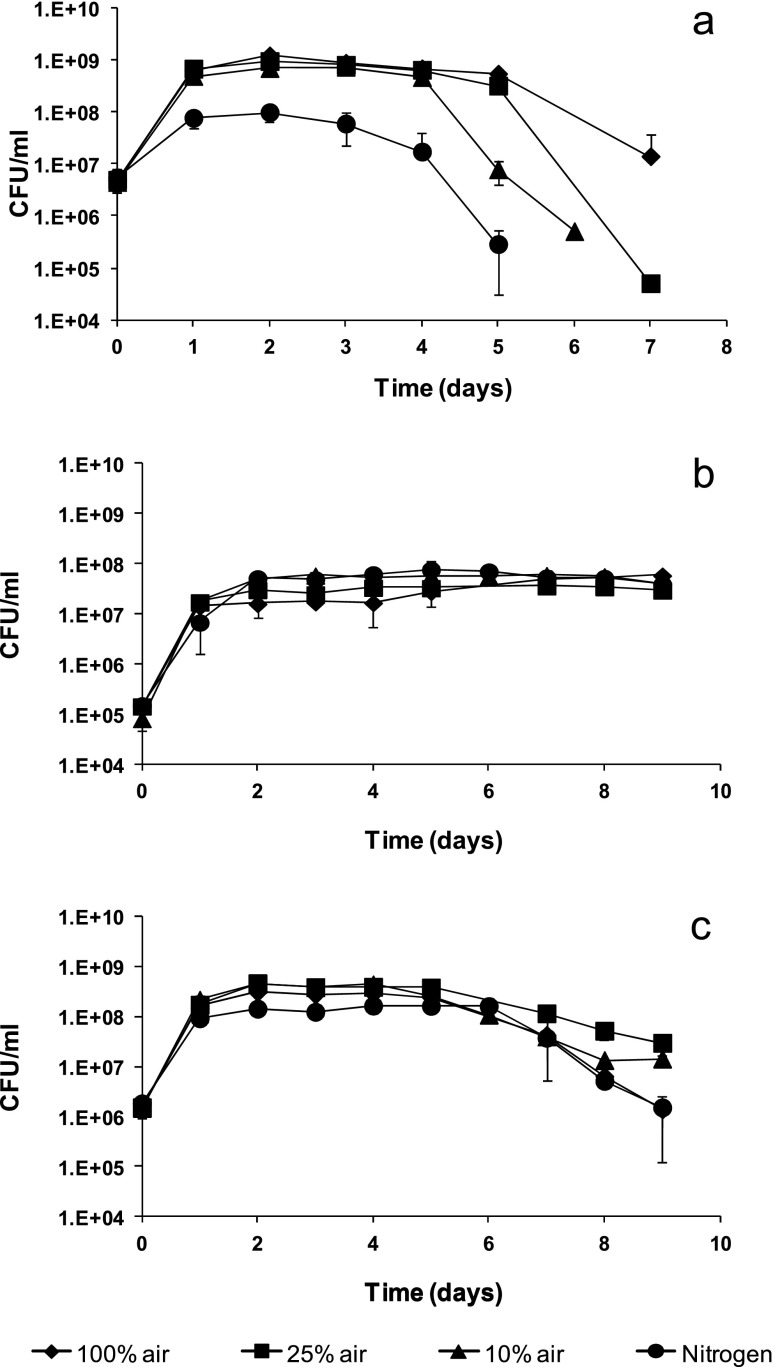
Viable cell counts for fermentations sparged with different gas mixtures, in the conditions described in the text, for **a** *M. pulcherrima* in cultures inoculated with *M. pulcherrim* + 10 % *S. cerevisiae*; **b** *S. cerevisiae* in cultures inoculated with *M. pulcherrima* + 10 % *S. cerevisiae*; or **c** *S. cerevisiae* pure cultures

In mixed cultures, *M. pulcherrima* was always replaced by *S. cerevisiae* by the end of the fermentation (Fig. 2). This replacement took place later for cultures sparged with air (around day 7) than for those sparged with nitrogen (around day 4). Indeed, growth of *M. pulcherrima* was dramatically restricted under nitrogen sparging, even in pure culture (Fig. 1). For oxygenated cultures, decay of this species was slower with increasing proportion of air during the gassing step, despite having reached similar maximum viable cell numbers. Growth of *S. cerevisiae* in these mixed cultures was very similar for all gassing conditions (Fig. 2). Maximum colony forming unit (CFU) counts fell below those of *M. pulcherrima*, except for the most anaerobic condition, but remained constant until the end of the fermentation process, in contrast to the relatively quick decrease in *M. pulcherrima* viability (Fig. 2). Viable counts for pure *S. cerevisiae* cultures were generally higher than in mixed cultures (Fig. 2).

### Dissolved oxygen

**Fig. 3:**
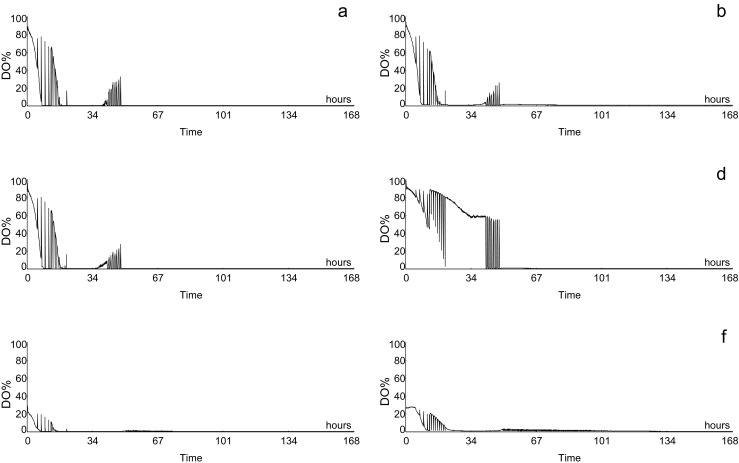
Evolution of dissolved oxygen levels (expressed as % saturation with air) in fermentations sparged with 100 % air (**a–d**) or 25 % air (**e–f**) in the conditions described in the text for different combinations of yeast strains in the inoculum: *M. pulcherrima* (**a**); *S. cerevisiae* (**d**, **f**); *M. pulcherrima* + 10 % *S. cerevisiae* (**c**, **e**); *M. pulcherrima* + 1 % *S. cerevisiae* (**b**)

We monitored DO levels during fermentation experiments as an indicator of the potential risk of oxidation of grape must components. DO levels during the first 48 h were absolutely dependent of the composition of the gas used to sparge the culture but also strongly dependent on the strain used. For all cultures inoculated with *M. pulcherrima* (either pure or in combination with *S. cerevisiae*), DO gradually dropped and stabilized around zero, even between 24 and 40 h, when sparging was continuous (Fig. 3). In contrast, cultures inoculated with *S. cerevisiae* alone showed DO levels above 50 % (i.e. about 3.5 mg/L) for most of the time; apart from the short periods, sparging was stopped, especially from 40 to 48 h (Fig. 3). As expected, DO values were clearly lower when 25 % instead of 100 % air was used (Fig. 3). Basal level was reached faster than in experiments performed with 100 % air and quicker for the mixed culture than for *S. cerevisiae* alone (Fig. 3). Not surprisingly, DO levels dropped to zero immediately after sparging was stopped, for any strain combination or gas composition.

## Discussion

### Growth

In agreement with our previous observations, *M. pulcherrima* showed better growth and sugar consumption under aerobic conditions (Table 1 and Fig. 1), as compared to anaerobiosis. However, after the switch to anaerobic conditions, the *M. pulcherrima* pure cultures were not able to complete fermentation. Slow or incomplete fermentations are common for non-*Saccharomyces* yeast strains and have been previously described for *M. pulcherrima* (Medina et al. 2012), although Sadoudi et al. (2012) recently described complete grape must fermentation by one strain of this species.

Under anaerobic conditions, faster fermentative metabolism confers a growth advantage to *S. cerevisiae* over *M. pulcherrima* (Fig. 1). However, *M. pulcherrima* is able to take better advantage of oxygen when it is available (Fig. 1). In mixed cultures, changes in the initial aeration regime had a stronger impact on the growth of *M. pulcherrima* than on *S. cerevisiae* (Fig. 2), resulting in a quick yeast strain replacement under anaerobic conditions. A competitive disadvantage for other non-*Saccharomyces* species in the absence of oxygen was previously described by Hansen et al. (2001). However, other mechanisms might also be involved in yeast-yeast competitive interactions, as shown by Nissen et al. (2003) or Bely et al. (2008) for different *S. cerevisiae*/*Torulaspora delbrueckii* strain combinations.

### Ethanol

Both *M. pulcherrima* and *S. cerevisiae* showed reduced ethanol yield in aerobic as compared to anaerobic cultures (Table 1). However, as expected for Crabtree-negative yeasts (Quirós et al. 2014), the extent of this reduction was higher for *M. pulcherrima*. The sudden removal of the air supply after 48 h resulted in increasing ethanol yields in the second part of the experiments for all culture conditions (Table 3). In agreement with our working hypothesis, as well as results by Giovanelli et al. (1996), a strong and significantly negative correlation between air concentration and the final yield of ethanol was found for both *S. cerevisiae* and mixed cultures (Table 5). Differences in final alcohol content between fully anaerobic or air sparged cultures were already relevant for pure *S. cerevisiae* cultures, 2.2 % (*v*/*v*), but were more important for mixed cultures, 2.9 % (*v*/*v*) and up to 3.7 % (*v*/*v*), when comparing anaerobic *S. cerevisiae* against aerated mixed cultures (Table 2). In addition to the shift from respiro-fermentative to pure fermentative metabolism, gradual replacement of *M. pulcherrima* by *S. cerevisiae* must also be contributing to the increase in final ethanol yield observed for mixed cultures in the second part of the experiments.

Contreras et al. (2014) described alcohol level reduction in non-aerated sequential inoculation cultures with one strain of *M. pulcherrima* and one of *S. cerevisiae*. This would be in agreement with the trend observed in Table 2 for nitrogen-sparged cultures. Other authors had also reported moderate alcohol level reductions by using non-*Saccharomyces* yeast strains, either pure or in co-culture, under non-aerated conditions (Ciani and Comitini 2006; Bely et al. 2008; Gobbi et al. 2013). Understanding the fate of carbon in those cases would require further analysis and improved knowledge on the metabolism of these non-conventional yeasts.

### Acetic acid and glycerol

The most noticeable impact of aeration on *S. cerevisiae* metabolism was on acetic acid production (Table 1). Moreover, a positive correlation was confirmed between acetic acid yield and oxygenation level (Table 5). This is in agreement with results by Giovanelli et al. (1996) who also found increased acetic acid yield under aerobic conditions (as compared to anaerobic) for the fermentation of commercial grape must. Likewise, Franzén (2003) found increased acetic acid yields with decreasing RQ values for this species. All these results are in contrast to those by Aceituno et al. (2012) who described acetic acid production to take place only under fully anaerobic conditions. Probably, the use of nitrogen-limited chemostat by Aceituno et al. (2012) is related to the differences they found in the pattern of acetic acid production, as compared to other authors.

Prior differences in the aeration regime had a huge impact on stage-specific acetic acid yields, even after sparging was completely stopped (Table 3). This can be related to differences in the biomass content or in the metabolic features of the cells, depending on the environmental conditions during the growth phase. Short-term aeration practices had been previously reported to influence global yeast physiology and fermentation kinetics (Valero et al. 2001; Fornairon-Bonnefond et al. 2003; Varela et al. 2012b). Interestingly, the use of mixed cultures conferred a clear advantage over pure *S. cerevisiae* to control volatile acidity, especially under intermediate oxygenation levels (Tables 2 and 4).

Dependence of glycerol yields on oxygen availability showed opposite trends for pure *S. cerevisiae* (negative correlation) or mixed cultures (positive correlation). The J-shaped graph obtained for mixed cultures seems to result from the differential effect of oxygen availability on the yeast strains used. By one side, according to data by Giovanelli et al. (1996) and results shown in Table 1, *S. cerevisiae* shows a greater glycerol yield under nitrogen sparging, being the most active strain under these conditions. By the other side, *M. pulcherrima* survival is favored under air sparging while it shows a higher glycerol yield than *S. cerevisiae* under all conditions (Table 1). Under intermediate oxygenation conditions, *S. cerevisiae* will tend to lower glycerol yield, while *M. pulcherrima* will not be so favored, resulting in lower glycerol yields than the extreme conditions. Metabolic interactions between the strains might also influence the different yields, as described for other *S. cerevisiae*/non-*Saccharomyces* strain combinations (Sadoudi et al. 2012; Milanovic et al. 2012).

### Dissolved oxygen

Oxygen consumption by *M. pulcherrima* in mixed cultures led to low DO levels during most of the aeration step, in contrast to pure *S. cerevisiae* cultures (Fig. 3). The use of air mixtures further helped to reduce DO levels. In all cases, DO fell to zero in the second, anaerobic step, as intended. Oxygen affinity of wine polyhenols has been determined to be about 1000 times lower than fermenting *S. cerevisiae* cells (Salmon 2006), so according to data shown in Fig. 3, we can expect a very low impact of the aeration regime described in this work on the oxidation of grape must components for fermentations driven by *M. pulcherrima* CECT12841 (alone or including up to 10 % of *S. cerevisiae* EC1118).

In summary, both the use of *M. pulcherrima* CECT12841 and air sparging during the first 48 h have a great impact on fermentation dynamics and the production of yeast metabolites during growth in natural grape must. Most of the observed effects could be explained in terms of differences in central carbon metabolism between the two yeast strains employed, either directly or through its influence on population dynamics. This work shows the potential of sugar respiration by non-*Saccharomyces* yeasts to help reduce alcohol levels in wine, as previously suggested (Gonzalez et al. 2013). More recently, Contreras et al. (2014) described decreased ethanol yields by sequential inoculation of *M. pulcherrima* and *S. cerevisiae*. Using different strains of both species and simultaneous inoculation, we showed the crucial role of oxygen availability and respiratory metabolism in order to reduce alcohol levels by up to 3.7 % (*v*/*v*) by the end of fermentation of a natural white grape must. By choosing and optimizing the appropriate gassing conditions (i.e. 25 % air), we managed to find a good balance between alcohol level reduction (2.2 % (*v*/*v*)), the increase in volatile acidity, mostly associated to growth of *S. cerevisiae* under aerobic conditions (below 0.35 g/L), and levels of dissolved oxygen during the process (most time being almost undetectable). However, we should keep in mind that practical constraints will be different between the industrial and the laboratory setup. For example, intermediate aeration levels were attained in this work by using gas mixtures (for operational reasons), but they could be reached in an industrial setup by simply reducing the gas flow and avoiding agitation. Therefore, the implementation at the industrial level of a strategy to lower ethanol content of wine, based on the respiratory breakdown of sugars by non-*Saccharomyces* yeasts, poses an interesting challenge that would require further optimization, involving yeast species and strain selection, inoculation strategies, development of oxygenation and mixing conditions and devices, or fermentation nutrients.

## Electronic supplementary material

Below is the link to the electronic supplementary material.

Table S1 (PDF 102 kb)
