# Supplementary material for: The impact of oxygen on the final alcohol content of wine fermented by a mixed starter culture
Source: Appl Microbiol Biotechnol. 2015 Jan 13;99(9):3993–4003. doi: 10.1007/s00253-014-6321-3 (PMC4428804; doi:10.1007/s00253-014-6321-3)
Supplement: Supplementary file 1 — (PDF 102 kb) [file 253_2014_6321_MOESM1_ESM.pdf]

Title:

The impact of oxygen on the final alcohol content of wine fermented by a mixed starter culture.

Journal:

Applied Microbiology and Biotechnology

Authors:

Pilar Morales[a], Virginia Rojas[a,b], Manuel Quirós*[a] and Ramon Gonzalez[a]

Affiliations:

Instituto de Ciencias de la Vid y del Vino (CSIC, Universidad de la Rioja, Gobierno de La Rioja). Logroño, La Rioja, Spain[a]. Facultad de Ciencias Químicas. Universidad de Autónoma de San Luis Potosí. Mexico[b].

*Present address: Evolva Biotech A/S. Copenhagen Ø. Denmark.

Corresponding authors:

Ramon Gonzalez, ramon.gonzalez@csic.es, or Pilar Morales, pilar.morales@icvv.es

Table S1. Gassing pattern (gas valve status) employed in all the experiments after the initial assay.

Time (h:min)	Gas valve status
0:00	0
1:58	1
5:00	0
6:55	1
7:00	0
8:55	1
9:00	0
10:55	1
11:00	0
11:55	1
12:00	0
12:10	1
13:00	0
13:10	1
14:00	0
14:10	1
15:00	0
15:10	1
16:00	0
16:10	1
17:00	0
17:10	1
18:00	0
18:10	1
19:00	0
19:10	1
20:00	0
20:10	1
41:00	0
41:30	1
42:00	0
42:30	1
43:00	0
43:30	1
44:00	0
44:40	1
45:00	0
45:40	1
46:00	0
46:40	1
47:00	0
47:40	1
48:00	0
